# Nose-to-brain delivery of self-assembled curcumin-lactoferrin nanoparticles: Characterization, neuroprotective effect and *in vivo* pharmacokinetic study

**DOI:** 10.3389/fbioe.2023.1168408

**Published:** 2023-03-27

**Authors:** Linghui Li, Liwei Tan, Qian Zhang, Yushan Cheng, Yayuan Liu, Rui Li, Shuguang Hou

**Affiliations:** ^1^ State Key Laboratory of Southwestern Chinese Medicine Resources, School of Pharmacy, Chengdu University of Traditional Chinese Medicine, Chengdu, Sichuan, China; ^2^ Sichuan Purity Pharmaceutical Co. Ltd., Chengdu, Sichuan, China

**Keywords:** curcumin-lactoferrin nanoparticles, nose-to-brain, neuroprotective effect, Aβ_25-35_, pharmacokinetic

## Abstract

Curcumin (CUR) is a natural polyphenol extract with significant antioxidant and anti-inflammatory effects, which indicates its great potential for neuroprotection. Lactoferrin (LF), a commonly used oral carrier and targeting ligand, has not been reported as a multifunctional nanocarrier for nose-to-brain delivery. This study aims to develop a nose-to-brain delivery system of curcumin-lactoferrin nanoparticles (CUR-LF NPs) and to further evaluate the neuroprotective effects *in vitro* and brain accumulation *in vivo*. Herein, CUR-LF NPs were prepared by the desolvation method with a particle size of 84.8 ± 6.5 nm and a zeta potential of +22.8 ± 4.3 mV. The permeability coefficient of CUR-LF NPs (4.36 ± 0.79 × 10^−6^ cm/s) was 50 times higher than that of CUR suspension (0.09 ± 0.04 × 10^−6^ cm/s) on MDCK monolayer, indicating that the nanoparticles could improve the absorption efficiency of CUR in the nasal cavity. Moreover, CUR-LF NPs showed excellent protection against Aβ_25-35_-induced nerve damage in PC12 cells. *In vivo* pharmacokinetic studies showed that the brain-targeting efficiency of CUR-LF NPs *via* IN administration was 248.1%, and the nose-to-brain direct transport percentage was 59.7%. Collectively, nose-to-brain delivery of CUR-LF NPs is capable of achieving superior brain enrichment and potential neuroprotective effects.

## 1 Introduction

As a class of typical central nervous system diseases, neurodegenerative diseases such as Alzheimer’s disease (AD), Parkinson’s disease (PD), and Huntington’s disease (HD) are characterized by high incidence and disability rates and are incurable. With the development of an aging population, such chronic diseases place a heavy burden on families and society (Miksys and Tyndale, 2010; Budd Haeberlein and Harris, 2015; Dugger and Dickson, 2017). Unfortunately, due to the complex pathogenesis of neurodegenerative diseases, current drug treatments have not shown satisfactory pharmacodynamic effects (Tofaris and Buckley, 2018). In addition, oral administration is a common route for most neurotherapeutic drugs, whereas the physiological protective effect of the blood-brain barrier (BBB) greatly limits drug exposure in the brain and further reduces the therapeutic effects (Patel et al., 2019; Dou et al., 2021). Meanwhile, excessive systemic exposure with oral administration may lead to intolerable toxicity and various side effects in the gastrointestinal tract and even in other organs (Lauzon et al., 2015). Intranasal (IN) administration has been considered an alternative route to oral administration for the delivery of neurotherapeutic drugs. Based on the better clarification of the physiological structure of the nasal cavity, it has been reported that active pharmaceutical ingredients (APIs) could be transported directly to the brain by bypassing the BBB *via* the trigeminal and olfactory regions (Agrawal et al., 2018; Crowe et al., 2018). Moreover, the respiratory region contains a large number of blood vessels, and APIs could be easily absorbed into the systemic blood circulation without enterohepatic circulation to avoid the first-pass effect (Erdo et al., 2018).

**FIGURE 1:**
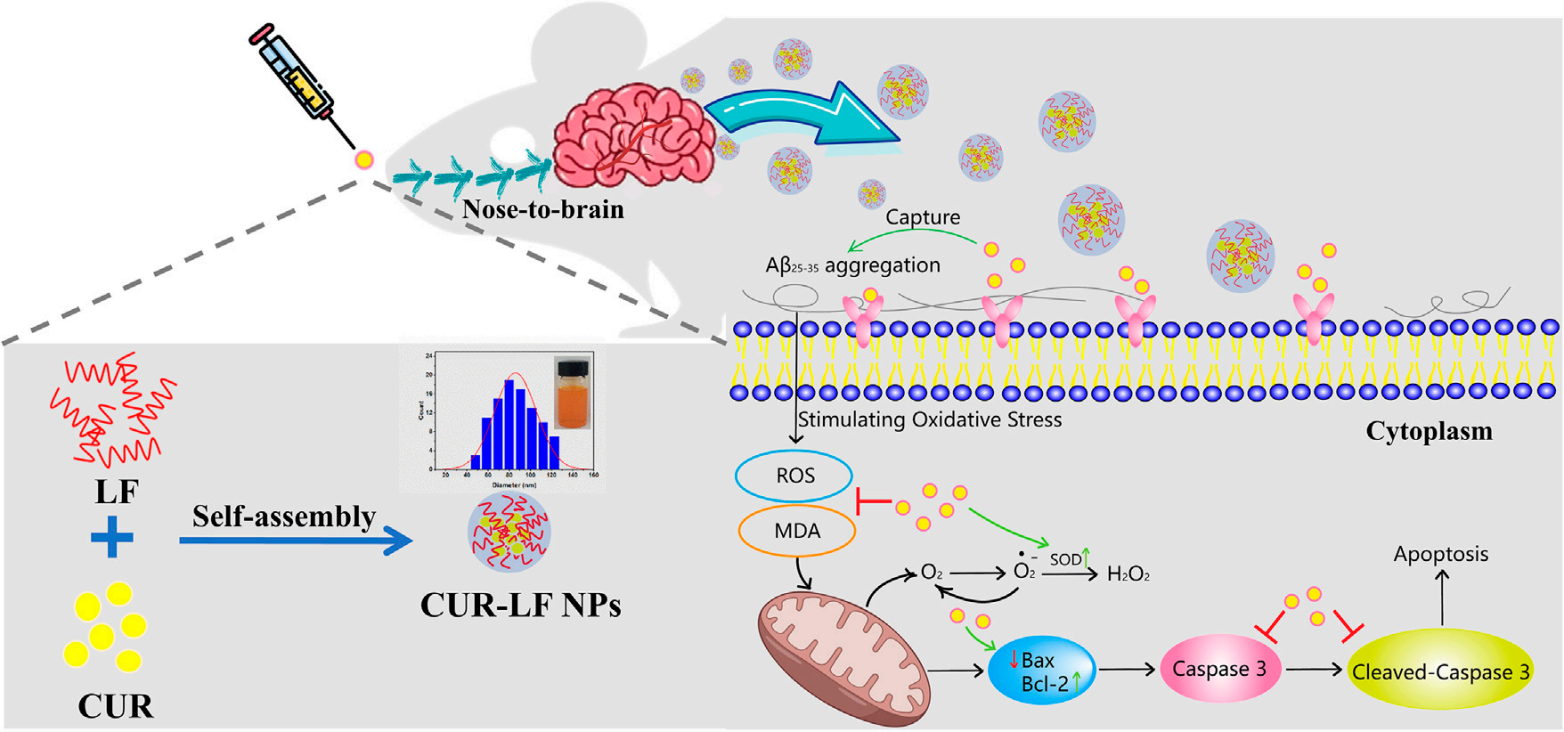
Schematic illustration of the preparation of curcumin-lactoferrin nanoparticles (CUR-LF NPs) and to evaluate the prospective and feasibility for neuroprotection *via* intranasal administration.

An increasing number of researchers believe that safe and mildly effective natural extracts, including but not limited to huperzine A, curcumin, hydroxysafflor yellow A, and resveratrol, are more beneficial for the long-term control and alleviation of chronic and persistent neurodegenerative diseases (Di Paolo et al., 2019). Among them, curcumin (CUR) is a natural polyphenolic compound that is extracted from the rhizomes of Zingiberaceae plants. It is widely used as a food additive and spice in India, where the incidence of AD is low (Reddy et al., 2018). It has been reported that CUR plays an important neuroprotective role in the early pathogenic stages of neurodegenerative diseases through antioxidant and anti-inflammatory effects (Vaz et al., 2017). However, similar to most natural compounds, the hydrophobic property, low permeability, short biological half-life, and difficulty in penetrating the BBB of CUR limit its further application in neurological diseases (Del Prado-Audelo et al., 2019; Zhang et al., 2020). Considering the properties of CUR, nanotechnology-based delivery systems have been recommended by numerous researchers for such complex natural compounds (Liu et al., 2013; Piazzini et al., 2019; Qizilbash et al., 2022). Among all biological nanomaterial candidates, lactoferrin (LF) is an 80 kDa monomeric glycoprotein belonging to the transferrin (TF) family (Wang et al., 2019). As a type of endogenous protein in the human body, LF is recognized to have not only high biological safety, but also to bind to TF and LF receptors that are highly expressed in brain endothelial cells (Sabra and Agwa, 2020). Therefore, it is commonly used as a ligand to modify nanoformulations to improve their efficiency in passing through the BBB (Nasr, 2016; Jiang et al., 2019; Agwa and Sabra, 2021; Kim et al., 2022). In addition, the excellent ability of LF as a drug carrier cannot be ignored. LF has been used as an oral carrier to cargo small molecule APIs (Li et al., 2018; Luo et al., 2021). As an amphipathic protein, the hydrophobic regions of LF provide suitable binding sites for insoluble APIs (Yan et al., 2017). Moreover, some studies have indicated that LF has a neuroprotective function (Zakharova et al., 2018; Elzoghby et al., 2020). It has been reported that LF could inhibit cognitive decline in AD mice by activating the expression of ADAM10 in the ERK1/2-CREB and HIF-1α signaling pathways *via* IN administration (Guo et al., 2017). As a consequence, LF could not only be used as a nose-to-brain nanocarrier and targeting ligand to promote efficient brain enrichment of CUR, but also play a synergistic neuroprotective effect with CUR. As shown in Figure 1, in this study, self-assembled CUR-LF NPs were prepared, and the neuroprotective mechanism of the nanoparticles on Aβ_25-35_-induced damage in PC12 cells was explored by antioxidant and anti-apoptosis analysis. We further studied the brain accumulation of CUR-LF NPs *via* IN administration to confirm their prospect and feasibility in neuroprotection.

## 2 Materials and methods

### 2.1 Materials, cells, and animals

CUR was purchased from Chengdu McLean Biotechnology Co., Ltd (Analytical Reagent, Chengdu, China). LF was purchased from Shanghai Yuanye Biotechnology Co., Ltd (95%, Shanghai, China). CUR standard (98.5%) and magnolol standard (98.5%) were purchased from Chengdu Pusi Biotechnology Co., Ltd (Chengdu, China). Aβ_25-35_ and DMSO were obtained from Sigma-Aldrich (Saint Louis, MO, United States). The antibody against β-tubulin and 4,6-diamidino-2-phenylindole (DAPI) were obtained from Beyotime Biotechnology Co., Ltd (Shanghai, China). The antibodies against Bax (#ab32503) and Bcl-2 (#ab196495) were obtained from Abcam (Cambridge, United Kingdom). The antibody against Caspase 3/p17/p19 (66470-2-lg) was obtained from proteintech^®^ (Chicago, United States). Goat Anti-Rabbit IgG H&L (HRP) and Goat Anti-Mouse IgG H&L (HRP) were obtained from Invitrogen (Carlsbad, CA, United States). Ultrapure water comes from the Milli-Q system (Millipore, Bedford, MA, United States). All solvents including glacial acetic acid, acetonitrile, formic acid, methanol, and absolute ethanol were HPLC grade and were obtained from Chengdu Cologne Chemical Co., Ltd. (Chengdu, China).

MDCK cells were purchased from Beina organisms (Beijing, China), and PC12 cells were kindly provided by Southwest Jiaotong University (Chengdu, China). The above cells were cultured in Dulbecco’s modified Eagle medium (DMEM), supplemented with 10% (v/v) fetal bovine serum (FBS), 1% (v/v) penicillin-streptomycin solution, and 1% (v/v) glutamine (Invitrogen). All reagents were freshly prepared, and free CUR was dissolved in DMSO and diluted more than 100 times with DMEM (containing 5% FBS) as the diluent.

Male Sprague-Dawley rats (SPF, 200–220 g) were purchased from Hunan Slake Jingda Laboratory Animal Co., Ltd (Hunan, China; permits SCXK 2019-0004). The protocol of animal experiments was approved by the Experimental Animal Ethics Committee of Chengdu University of Traditional Chinese Medicine. Rats were reared under standard conditions and had free access to food and distilled water.

### 2.2 Preparation of CUR-LF NPs

CUR-LF NPs were prepared by the desolvation method. Briefly, 1 mL of CUR ethanol solution (6 mg/mL) was added dropwise (1 mL/min) to 16 mL of LF solution (dispersed in ultrapure water, 3 mg/mL) under magnetic stirring (400 rpm). The mixture was homogenized using a probe sonicator at 100 W power, on for 2 s and off for 3 s, and cycled for 2 min. CUR has poor water solubility (11.33 ± 0.73 ng/mL) and the unencapsulated CUR exists in solution as microcrystalline material. Therefore, free CUR was separated by centrifugation precipitation (4,000 rpm, 10 min). The supernatant was filtered through a 0.22 μm filter to obtain CUR-LF NPs solution.

### 2.3 Characterization of CUR-LF NPs

#### 2.3.1 Drug encapsulation capability

Encapsulation efficiency (EE, %) and drug loading (DL, %) of CUR-LF NPs were evaluated by HPLC (Agilent 1260 Infinity II, Agilent, Santa Clara, CA, United States) using an Agilent ZORBAX Eclipse Plus C18 (150 mm × 4.6 mm, 3.5 μm) chromatographic column at a detection wavelength of 430 nm. The mobile phase was 4% glacial acetic acid/acetonitrile (52/48, v/v) with a flow rate of 1 mL/min. The EE (%) and DL (%) were calculated by the following equations.

$$EE\;\left(\%\right)=\frac{Weight\;of\;Curcumin\;in\;CUR‐LF\;NPs}{Initial\;dosage\;of\;Curcumin}$$ (1)

$$DL\;\left(\%\right)=\frac{Weight\;of\;Curcumin\;in\;CUR‐LF\;NPs\;Lyophilized\;Powder}{Weight\;of\;CUR‐LF\;NPs\;Lyophilized\;Powder}$$ (2)

#### 2.3.2 Particle size, zeta potential, and micromorphology

The mean particle size, polydispersity index (PDI), and zeta potential of CUR-LF NPs were conducted using a NanoBrook 90 Plus PALS instrument (Brookhaven Instruments Corporation, United States). The results were obtained from more than 3 parallel samples.

The micromorphology of CUR-LF NPs was observed by transmission electron microscopy (TEM) (HT7800, HITACHI, Japan). The observation was performed by applying a drop of CUR-LF NPs on a special copper mesh, followed by negative staining with 2.0% (w/v) sodium phosphotungstate.

#### 2.3.3 Circular dichroism (CD)

UV circular dichroism (CD) spectra of the native LF and CUR-LF NPs were recorded in the range 190–250 nm with 0.25 mg/mL protein by a Chirascan qCD (Applied Photophysics Ltd., United Kingdom). The CD spectrum was the average of 3 measurements at a test speed of 100 nm/min, 0.2 nm resolution, 1.0 nm bandwidth, and nitrogen as shielding gas (Li et al., 2018). The collected data was analyzed using Dichroweb (Circular Dichroism Website http://dichroweb.cryst.bbk.ac.uk) to obtain the secondary structure of LF.

#### 2.3.4 X-ray diffraction (XRD)

X-ray diffraction (XRD) measurements of CUR, LF, CUR/LF physical mixture, and CUR-LF NPs at 6°/min scanning from 5° to 40° (2θ) at 40 kV and 30 mA using the XRD instrument (DX-2700 BH, Dandong Haoyuan Instrument Co., Ltd. China).

#### 2.3.5 Differential scanning calorimetry (DSC)

Calorimetric analyses were determined using differential scanning calorimetry (DSC) instrument (DSC 3+, Mettler Toledo Ltd. Switzerland). The aluminum pans containing approximately 3–5 mg of samples were heated from 30°C to 300°C at a rate of 10°C/min under a nitrogen environment.

#### 2.3.6 Stability of CUR-LF NPs

To simulate the stability of CUR-LF NPs *in vivo*, we mixed CUR-LF NPs with PBS (pH 7.4), DMEM, simulated nasal fluid (SNF, pH 6.0), and artificial cerebrospinal fluid (ACSF, pH 7.4) at a volume ratio of 1:9 and incubated the mixture in a constant temperature shaker (100 rpm) at 37°C. CUR is extremely insoluble in water, thus, the leaked free CUR will be precipitated at the bottom of the test tube after the nanoparticles rupture. A total of 200 μL of supernatant containing CUR-LF NPs was collected, and the content of CUR-LF NPs was determined by HPLC at 0, 2, 4, 8, 12, and 24 h. Then, the nanoparticles’ stability rate was calculated (the stabilization ratio of CUR-LF NPs at 0 h was 100%).

### 2.4 *In vitro* delivery evaluation

#### 2.4.1 *In vitro* release studies

A discriminatory and rugged dialysis method was developed for CUR-LF NPs to measure the drug release profiles (Li et al., 2022). Briefly, 1 mL of CUR solution (in 50% DMSO solution, v/v), CUR suspension (in 0.5% CMC-Na solution, w/v), and CUR-LF NPs with a concentration of 200 μg/mL were added into dialysis bags (MWCO 8000–14000 Da). They were immersed in 30 mL of release media composed of PBS buffer (pH 7.4) containing 30% ethanol (v/v) and placed in a constant-temperature shaker incubator (100 rpm) at 37°C. The release system was completely replaced with 30 mL of prewarmed, fresh media at 0.25, 0.5, 1, 2, 4, 6, 8, 12, and 24 h. Release tests for each sample were performed in triplicate. The concentration of CUR was determined by HPLC, and the cumulative release (*Q*, %) was obtained according to the following equation.

$$Q=[(V.\sum_{i=1}^{n}C_{i})/M]\times 100$$ (3)

Where V is the volume of the release media; Ci is the curcumin concentration measured at each time point; M is the initial dosage.

#### 2.4.2 MDCK monolayer penetration studies

First, the cytotoxicity of CUR formulations incubated with MDCK cells for 4 h was measured using Cell Counting Kit-8 (CCK-8, Dojindo Molecular Technologies, Inc. Rockville, MD, United States) (Supplementary Figure S1). MDCK cells (1.8×10^5^/well) were seeded onto the apical chamber of cell culture inserts (1.12 cm^2^, 3 μm, polycarbonate membrane; Corning, New York, United States) in a 12-well plate and cultured for 3–5 days. The transepithelial electrical resistance (TEER) of each insert was monitored by an epithelial volt-ohm meter (MERS00002, Millipore) to reach higher than 0.9 kΩ cm^2^ prior to the penetration studies. Then, 0.5 mL of 100 μg/mL CUR solution (containing 1% DMSO), 0.5% carboxymethyl cellulose sodium (CMC-Na) CUR suspension, and CUR-LF NPs were added to the apical chamber, and 1.5 mL of HBSS solution (containing 0.05% Tween) was added to the basolateral chamber of the inserts. A 200 μL solution was collected from the basolateral chamber at 0.25, 0.5, 1, and 2 h, and the corresponding solution was replenished. The integrity of the MDCK monolayer cell membrane was determined by monitoring TEER changes throughout the process. The concentration of CUR in the basolateral chamber was determined by HPLC, and the apparent permeability coefficient (P_app_, cm/s) was obtained according to the following equation.

$$P_{app}=\left(dQ/dt\right)/C_{o}.A$$ (4)

Where d*Q*/d*t* is the permeability rate (μg/s); C_0_ is the initial curcumin concentration of the apical chamber (μg/mL); A is the polycarbonate membrane area (cm^2^).

### 2.5 *In vitro* efficacy evaluation of CUR-LF NPs in PC12 cells

#### 2.5.1 Cellular uptake

Fluorescence microscopy (CKX53SF, Olympus Corporation, Japan) was used to qualitatively observe the cellular uptake of free CUR and CUR-LF NPs based on the spontaneous fluorescence of CUR (λEx/λEm was 425/530 nm). PC12 cells (4×10^5^/well) were seeded into 6-well plates and cultured overnight. Then, free CUR and CUR-LF NPs (20 μM) with or without LF pretreatment were added and incubated for 60 min. After three washes with prechilled PBS, the cells were fixed in paraformaldehyde for 15 min. The nucleus was stained with DAPI for 5 min and then observed by fluorescence microscopy (Bonaccorso et al., 2020). Images were captured using the BioHD-C20 imaging system.

Quantitative analysis of cellular uptake was performed by HPLC. PC12 cells were treated with free CUR and CUR-LF NPs (20 μM) and incubated for 5, 30, 60, 120, and 240 min. The cells were collected by cell scraper and centrifugation (300 × g for 5 min at 4°C), cell lysis buffer containing the protein inhibitor cocktail (Merck) was added and lysed on ice for 30 min. Then, 4× volume of methanol was added to precipitate protein and extract CUR. HPLC analysis was performed on the supernatant. The protein concentration was determined by bicinchoninic acid assay (BCA assay, Beyotime). Quantitative cellular uptake was calculated by CUR concentration/protein content (μg/mg protein).

#### 2.5.2 Cytotoxicity assays

CCK-8 was used to determine the safe dose range of CUR-LF NPs in PC12 cells. The cells were seeded into 96-well plates (1×10^5^ cells/mL, 100 μL/well, 3 duplicate wells per group) and incubated overnight. Then, free CUR and CUR-LF NPs (0, 1, 10, 20, 30, and 50 μM) were added and incubated for 24 h. The negative control groups were cultured with the corresponding blank solvent. Each well was replaced with fresh DMEM and 10% CCK-8 in place of the previous medium, followed by incubation for 1–4 h. Pure CCK-8 medium was used as a blank well (Qian et al., 2018). The absorbance value of CCK-8 was detected at 450 nm with an automatic microplate reader (VICTOR NIVO, PerkinElmer, United States).

$$Cell\;viability\;\left(\%\right)=\frac{Mean\;absorbance\;of\;experimental\;well-average\;absorbance\;of\;the\;blank}{Mean\;absorbance\;of\;control\;well-average\;absorbance\;of\;the\;blank}\times 100$$ (5)

#### 2.5.3 Protective effects of CUR-LF NPs on Aβ_25-35_-induced PC12 cells

Briefly, 1 mg of Aβ_25-35_ was evenly dissolved in 200 μL of DMSO, and then 743 μL of DPBS was added to obtain a 1 mM stock solution. The aliquoted stock solution was incubated at 37°C for 7 days to allow Aβ_25-35_ to aggregate and age prior to the experiment. The cytotoxic effect of Aβ_25-35_ (1, 10, 25, and 50 μM) in PC12 cells with 24 and 48 h induction times were investigated. The treatment groups (free CUR, LF NPs, LF NPs + free CUR, and CUR-LF NPs) were added to the medium after 24 h of Aβ_25-35_ induction, and then co-incubated with Aβ_25-35_ for another 24 h.

#### 2.5.4 Oxidative stress assays

Oxidative stress was evaluated by detecting the levels of reactive oxygen species (ROS), superoxide dismutase (SOD), and malondialdehyde (MDA). Among them, ROS was detected using a DCFH-DA probe (Beyotime). The fluorescence of DCF was detected by an automatic microplate reader (λEx/λEm was 488/525 nm), and the results were expressed as relative fluorescence intensity (the control group was 100%). In addition, SOD and MDA were determined according to the protocol of the corresponding assay kits (Beyotime), and the protein concentration was determined as described above. The results of SOD and MDA were expressed as U/mg protein and μM/mg protein, respectively.

#### 2.5.5 Cell apoptosis analysis

Cell apoptosis is commonly detected using an Annexin-V-FITC/PI apoptosis detection kit (Vazyme Biotechnology Co., Ltd. Nanjing, China). PC12 cells were treated according to the protocol of the apoptosis kit. The samples were analyzed by flow cytometry (EXFKOW-206, DAKEWE Biotech Co., Shenzhen, China) within 1 h.

#### 2.5.6 Western blotting

Equal protein samples after heat denaturation were separated by SDS-PAGE (12%) at 80 V and transferred to 0.45 μm PVDF membranes. The membranes were blocked and incubated with antibodies. Visualization of the bands was performed using the ECL luminescence kit, and grayscale analysis was performed using ImageJ software (National Institute of Health, Bethesda, MD, United States). β-tubulin was used as an internal reference to facilitate data normalization.

### 2.6 *In vivo* studies of CUR-LF NPs

#### 2.6.1 Grouping and administration of animals

Rats were randomized into 3 groups of 18 animals each (n = 3, at each time point). The experimental animals were anesthetized with 2% pentobarbital sodium intraperitoneally prior to administration. Rats in Groups 1 and 2 were administered 0.5% CMC-Na CUR suspension and CUR-LF NPs *via* the IN administration route (100 μL, 10 mg/kg), respectively. Group 3 was administered CUR-LF NPs *via* tail vein injection (0.5 mL, 10 mg/kg). The rats were sacrificed at predetermined time points at 0.25, 0.5, 1, 2, 4, and 8 h. Blood was taken from the abdominal aorta, and the whole brain was rapidly dissected and washed with saline. Subsequently, the plasma samples were collected by centrifugation (4,000 rpm, 10 min). Saline was added to the brain tissue (brain tissue:saline ratio 1:2 w/w) and homogenized using a SCIENTZ-24 High Throughput Tissue Grinder (Ningbo Scientz Biotechnology Co., Ltd. Ningbo, China) at 2000 rpm for 30 s. Samples were stored at −20°C, processed, and analyzed within 3 days.

#### 2.6.2 Processing of samples and chromatographic conditions

Briefly, 20 μL of magnolol methanol solution was spiked into the samples as an internal standard (IS, 10 μg/mL). Then, 0.8 mL of acetonitrile was added to 200 μL of plasma, and 1 mL of ethyl acetate and n-hexane (1:1, v/v) was added to 200 μL of brain homogenate. Each sample was vortexed and centrifuged (10,000 rpm for 10 min at 4°C) to precipitate protein and extract CUR and magnolol. The supernatant was evaporated to dryness at 40°C using a dry nitrogen blower (Shanghai Bilang Instrument Manufacturing Co., Ltd. Shanghai, China). The residue was dissolved in 200 μL of methanol and filtered through a 0.22 μm filter.

The concentration of CUR was analyzed by UPLC-MS/MS using a Thermo Scientific Vanquish™ Flex coupled to TSQ Fortis™ MS (Thermo, Massachusetts, United States) on a ZORBAX SB-C18 column (2.1 × 150 mm, 3.5 µm, Agilent). The mobile phase consisted of acetonitrile (A) and 0.1% aqueous solution of formic acid (B) at a flow rate of 0.2 mL/min according to the following time gradient elution program: 0–6 min, 50%–85% (A), 6–8 min, 85%–95% (A), 8–10 min, 95% (A). Sample analysis was performed by multiple reaction monitoring (MRM) in negative ion mode using m/z 367.138/173.06 for CUR and m/z 265.088/224.071 for magnolol as monitor ions.

#### 2.6.3 Pharmacokinetics parameters of CUR-LF NPs

The pharmacokinetic parameters were calculated by PK solver software (designed by China Pharmaceutical University, Nanjing, China) (Zhang et al., 2010) using the non-compartment model. Brain targeting efficiency (BTE, %) and nose-to-brain direct transport percentage (DTP, %) were calculated by the following equation (Hao et al., 2016):

$$BTE\;\left(\%\right)=\frac{\left(\frac{{AUC}_{Brain}}{{AUC}_{Plasma}}\right)IN}{\left(\frac{{AUC}_{Brain}}{{AUC}_{Plasma}}\right)IV}\times 100$$ (6)

$$DTP\;\left(\%\right)=\frac{{AUC}_{BrainIN}-\left(\frac{{AUC}_{BrainIV}}{{AUC}_{PlasmaIV}}\times {AUC}_{PlasmaIN}\right)}{{AUC}_{BrainIN}}\times 100$$ (7)

### 2.7 Statistical analysis

The results were presented as mean ± standard deviation. All data were analyzed using SPSS Statistics 23 software (Chicago, IL). Statistical analysis was performed using independent samples t-test and one-way ANOVA. *p* ^*^ < 0.05 and *p* ^**^ < 0.01 were considered statistically significant.

## 3 Results and discussion

### 3.1 Preparation of CUR-LF NPs

The preparation process of CUR-LF NPs was optimized in our previous work (Li et al., 2022). We found that the volume ratio of ethanol to water was the key factor affecting the formation of the nanoparticles. It has been reported that CUR relies primarily on hydrophobic and hydrogen bonding forces to bind to the hydrophobic region of LF (Araujo et al., 2020). Adding an appropriate amount of ethanol could alter the permittivity of the protein mixed solvent (Hansen et al., 2021), promote the unfolding of protein molecules and thus expose the hydrophobic nucleus (Halder and Jana, 2021), which is conducive to reducing the hydration of protein and increasing the binding efficiency with the hydrophobic CUR molecule. However, when the volume ratio of ethanol to water was greater than 1:4, nanoparticles could not be formed. Similar results were also reported by Gong et al. (Gong et al., 2015). A possible explanation for this might be that hydrogen bonds were formed between excessive ethanol and protein. The protein was denatured and precipitated due to the destruction of the original hydrogen bond structure inside the protein. As a result, stable CUR-LF NPs could be formed well when the volume ratio of ethanol to water was between 1:8 and 1:16, where the ratio of 1:16 showed better EE, particle size, and PDI. Therefore, it was selected for preparation in this study.

### 3.2 Characterization of CUR-LF NPs

The HPLC method for the determination of CUR concentration showed reasonable linearity in the concentration range from 0.76 to 141.84 μg/mL (*R* ^2^ = 0.9998) (Supplementary Figure S2). CUR-LF NPs showed an acceptable drug encapsulation capacity with EE and DL values of 91.2% ± 3.6% and 9.6% ± 0.8%, respectively.

**FIGURE 2:**
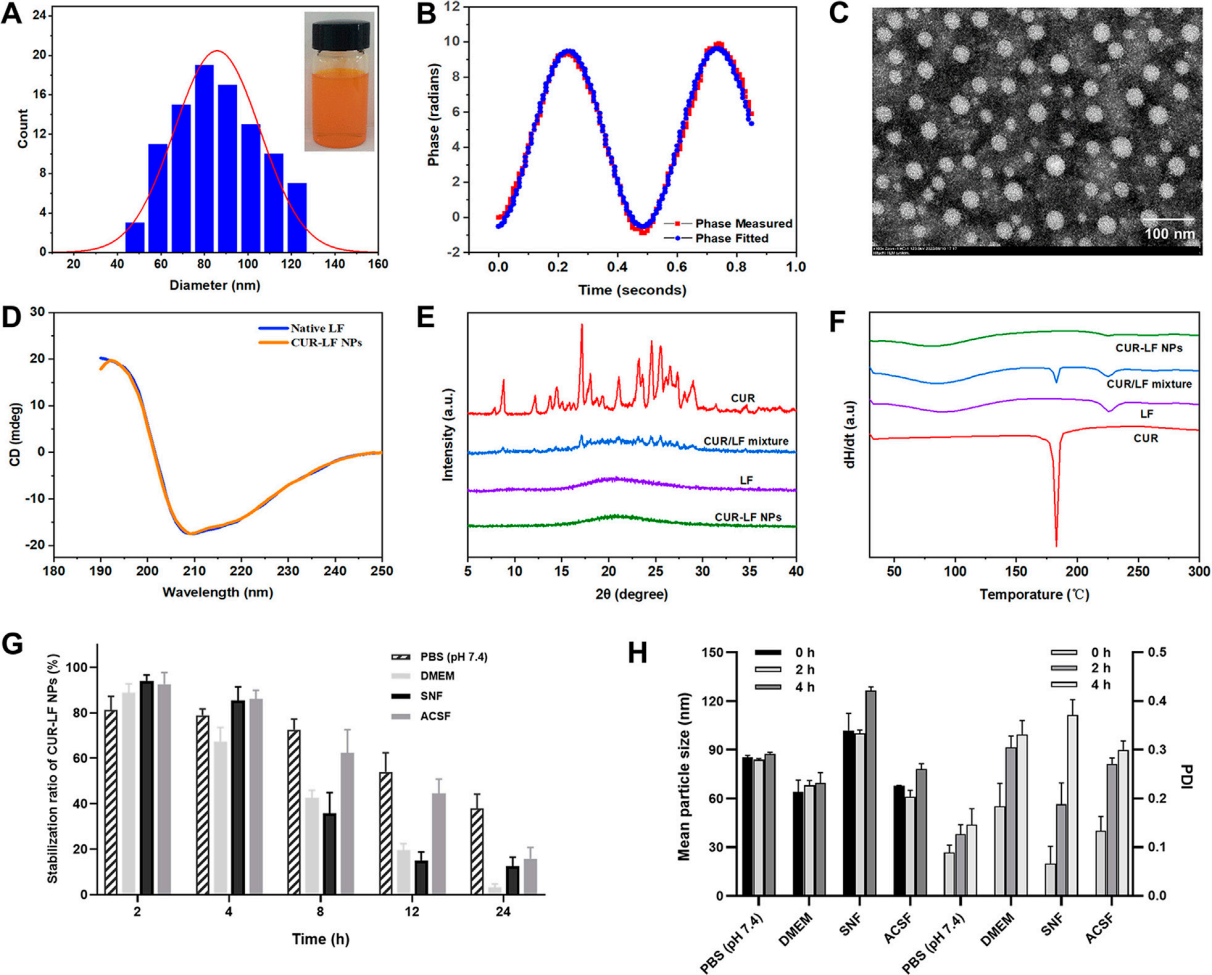
Characterization of CUR-LF NPs **(A)** Particle size distribution and appearance of CUR-LF NPs; **(B)** Zeta potential fitting diagram of CUR-LF NPs; **(C)** Transmission electron microscopy (× 100 000) of CUR-LF NPs; **(D)** Determination of secondary structure of native lactoferrin and CUR-LF NPs by circular dichroism; **(E)** X-ray diffractogram of curcumin, lactoferrin, curcumin/lactoferrin physical mixture, and CUR-LF NPs; **(F)** Differential scanning calorimetry of curcumin, lactoferrin, curcumin/lactoferrin physical mixture, and CUR-LF NPs; **(G)** Evaluation of the stabilization ratio of CUR-LF NPs incubated in PBS (pH 7.4), Dulbecco’s modified Eagle medium (DMEM), simulated nasal fluid (SNF), and artificial cerebrospinal fluid (ACSF) for 24 h; **(H)** Evaluation of the mean particle size and polydispersity index of CUR-LF NPs incubated in PBS (pH 7.4), DMEM, SNF, and ACSF for 4 h. Data were presented as mean ± SD (*n* = 3).

As shown in Figure 2A, CUR-LF NPs appeared as a clear yellow solution. The particle size of CUR-LF NPs was 84.8 ± 6.5 nm, and the PDI was 0.078 ± 0.016. The particle size was within the most suitable range (<200 nm) for nose-to-brain delivery with a uniform particle size distribution (Samaridou and Alonso, 2018). In addition, the TEM image (Figure 2C) showed that CUR-LF NPs were in a regular uniform spherical shape. The zeta potential was +22.8 ± 4.3 mV, and the phase measured and phase fitted curves almost coincided, which indicated that the measured data were reliable (Figure 2B).

CD is a widely used method for the detection of the secondary structure of proteins in the far ultraviolet range (190–250 nm) (He et al., 2016). As shown in Figure 2D, native LF exhibited the largest positive peak and negative peak in the wavelength ranges of 190–195 nm and 205–210 nm, respectively, which is mainly characteristic of α-helix (Liu et al., 2015). The spectrum of β-folded CD has a negative band in the wavelength range of 215–220 nm. The secondary structure contents of native LF and CUR-LF NPs were calculated using the Dichroweb website. There was no significant difference between them (*p* > 0.05), as shown in Table 1, indicating that the binding of CUR to LF with non-covalent bonds did not significantly affect the backbone hydrogen bond structure in LF, which was similar to the study by Araujo et al. (2020).

**TABLE 1:** Secondary structural contents of native lactoferrin and CUR-LF NPs. Data were presented as mean ± SD (n = 3).

	α-helix (%)	β-sheet (%)	β-bend (%)	Randomcoil (%)
Native LF	18.2 ± 1.1	33.3 ± 2.2	22.6 ± 2.1	26.5 ± 1.4
CUR-LF NPs	18.4 ± 0.8	34.2 ± 1.3	21.9 ± 1.2	26.0 ± 1.8

The XRD diffractograms of CUR, LF, CUR/LF physical mixture, and CUR-LF NPs were shown in Figure 2E. The strong signal characteristic diffraction peaks of crystalline CUR and the smooth XRD curve of amorphous LF could be observed. In addition, CUR and LF were physically mixed according to the preparation feeding ratio. The characteristic diffraction peaks of crystalline CUR were still observable, although the intensity was weakened. Finally, it was clear that the characteristic diffraction peaks of CUR in CUR-LF NPs disappeared, indicating that CUR was transformed into an amorphous state after being encapsulated with LF.

As shown in the DSC analysis (Figure 2F), the melting point of CUR was approximately 183°C, and the glass transition temperatures of LF were 89°C and 225°C. The specific endothermic peaks of CUR appeared in the physical mixture group at corresponding positions and disappeared in the CUR-LF NPs group. These phenomena could be explained by the fact that CUR was successfully encapsulated in the hydrophobic core of LF, rather than simply being physically mixed, which was consistent with the XRD analysis.

From Figures 2G, H, the stability magnitude of CUR-LF NPs in each culture system was PBS (pH 7.4) > ACSF > SNF > DMEM. Because nasal absorption is a rapid process, the stability of the nanoparticles in SNF ensures that CUR-LF NPs are absorbed by the nasal mucosa in a relatively complete nanostructure. Moreover, CUR-LF NPs showed more than 50% stability in ACSF within 12 h, which allows the nanoparticles to exert their effect in the brain to the greatest possible extent. Meanwhile, we found that the particle size of CUR-LF NPs did not significantly change within 4 h in each culture system, but the PDI showed an increasing trend.

### 3.3 *In vitro* release behaviors

**FIGURE 3:**
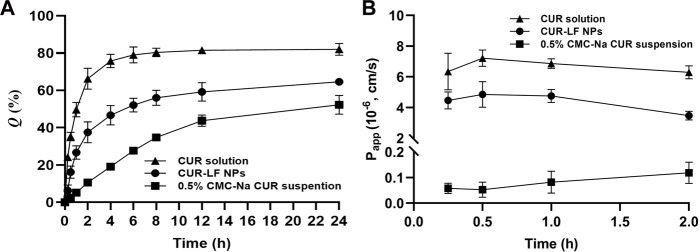
*In vitro* delivery evaluation **(A)** *In vitro* release of curcumin solution, 0.5% carboxymethyl cellulose sodium (CMC-Na) curcumin suspension, and CUR-LF NPs **(B)** The apparent permeability coefficient of curcumin solution, 0.5% CMC-Na curcumin suspension, and CUR-LF NPs on MDCK monolayer. Data were presented as mean ± SD (*n* = 3).

Dialysis methods are frequently used for the simulation of the *in vitro* release kinetics of nanoparticle-based drug delivery systems (Yu et al., 2019). As shown in Figure 3A, free CUR was rapidly released within the first 2 h, and the equilibrium between the solutions inside and outside the dialysis bag was almost achieved at 8 h with a cumulative release rate of 82.0% ± 3.2%. Compared to free CUR, the equilibrium was not achieved during the release process for both the CUR-LF NPs and CUR suspension, with much lower cumulative release rates of 64.6% ± 1.6% and 52.3% ± 5.0%, respectively. In comparison, the release profile of CUR from the nanoparticles was significantly higher than that from the suspension. The obtained release curves were fitted with different mathematical models, and it was found that they could be described well by the first-order model (Supplementary Table S1). There is no doubt that first-order processes are only dependent on the concentration of the dissolving substance (May et al., 2012). Therefore, the release rate of CUR from the nanocarriers was faster than the dissolution rate of CUR particles during release.

### 3.4 MDCK monolayer penetration studies

To evaluate the absorption and penetration of APIs in common *in vitro* cell models, some previous studies indicated that MDCK cells showed superior *in vitro* and *in vivo* correlation of *in vitro* permeability and *in vivo* nasal absorption in rats compared to Caco-2 and Calu-3 cells (Furubayashi et al., 2020). In addition, the MDCK cells formed tight junctions with a shorter molding time of 3 days and a high TEER value of over 0.9 kΩ.cm2, which is beneficial for the effective evaluation of nasal mucosal penetration efficiency of APIs in basic research. Therefore, the MDCK cell line was selected to establish a nasal mucosal model to investigate the *in vitro* penetration ability of different CUR formulations in this study. It is considered that APIs absorption through the nasal cavity requires rapid dissolution and passage through the mucus layer, which prevents the APIs from being removed from the absorption site by the nasal cilia, and then the APIs penetrate through the barrier of the epithelium, basement membrane, and lamina propria (He et al., 2020; Tai et al., 2022). As shown in Figure 3B, the insoluble CUR group was uniformly dispersed in a 0.5% CMC-Na suspension with a P_app_ of 0.09 ± 0.04×10^−6^ cm/s, which showed such a low permeability strength. CUR-LF NPs ameliorated the solubility of CUR, thus greatly improving the P_app_ (4.36 ± 0.79×10^−6^ cm/s) to a moderate permeability strength, which was very close to that of the CUR molecule in the solution state (6.68 ± 1.21×10^−6^ cm/s). APIs transport across the MDCK monolayer occurs mainly *via* paracellular and transcellular pathways. Due to the dense tight junctions between MDCK cells, the paracellular transport of CUR-LF NPs was greatly limited, and the CUR molecule was easier to transport *via* paracellular and transcellular pathways due to its low molecular weight and lipophilicity (Akel et al., 2020). As a result, the P_app_ of CUR solution was slightly higher than that of CUR-LF NPs.

### 3.5 Cellular uptake

**FIGURE 4:**
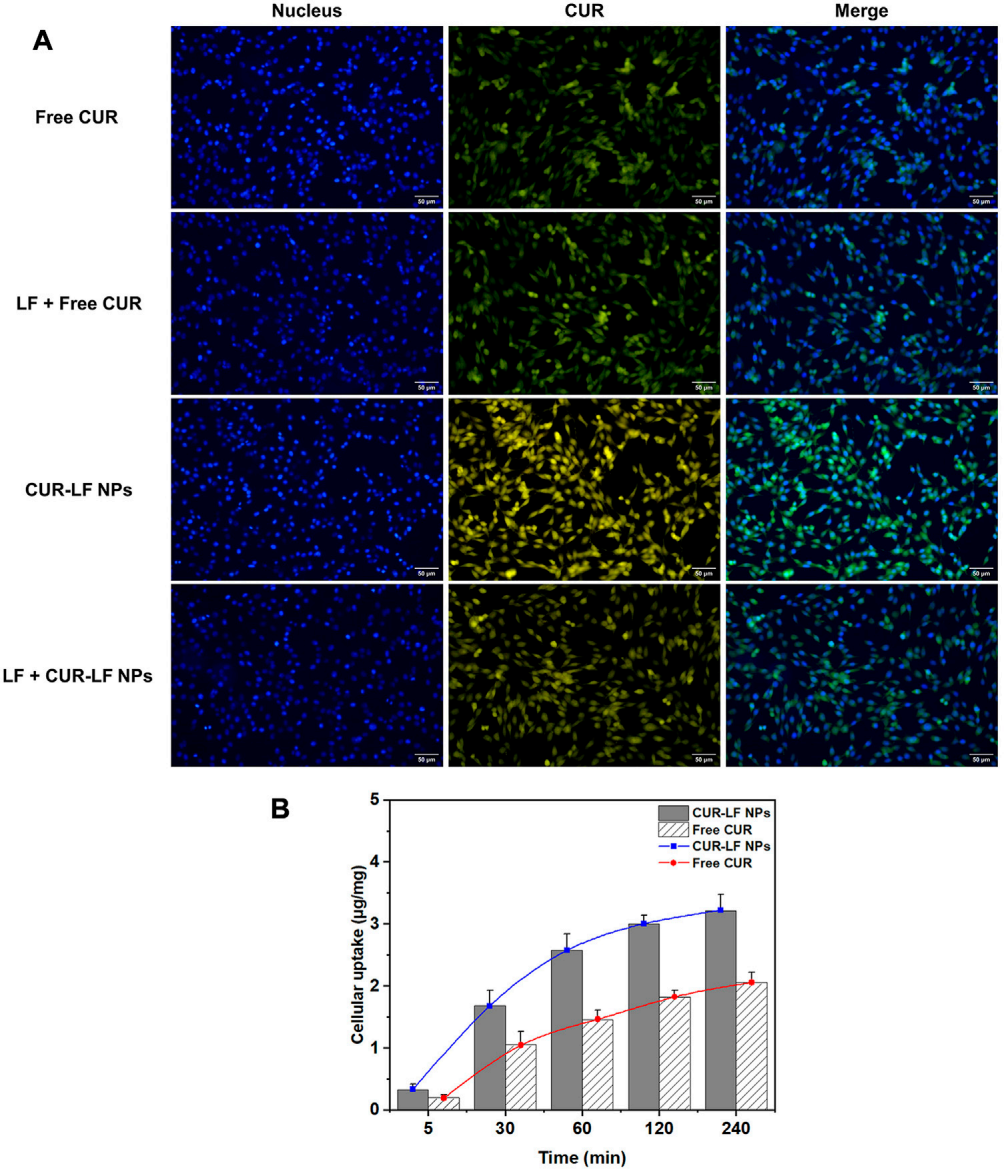
Cellular uptake of different curcumin formulations in PC12 cells **(A)** Fluorescence microscopy qualitative analysis of cellular uptake after incubation for 60 min with free curcumin and CUR-LF NPs with or without lactoferrin pretreatment at the concentration of 20 μM; **(B)** HPLC quantitative analysis of cellular uptake after incubation for 5, 30, 60, 120, and 240 min with the concentration of 20 μM. Data were presented as mean ± SD (*n* = 3).

PC12 cells were selected to analyze the cellular uptake of free CUR and CUR-LF NPs, as this cell line is widely used in neuroprotection mechanism research (Kwan et al., 2021). It is well known that increasing the cellular uptake efficiency is equivalent to effectively improving the therapeutic effect of APIs. Qualitative uptake was detected by fluorescence microscopy, as shown in Figure 4A. There was an interesting phenomenon that the fluorescence of free CUR was green, whereas that of CUR-LF NPs was yellow-green. We speculate that this occurred because CUR’s luminescent groups underwent subtle changes during binding to LF. This resulted in a shift in the emission wavelength and a further change in fluorescence. Similarly, a blue shift in the emission wavelength of CUR was found in Tween 80 micellar nanocurcumin prepared by Pinilla-Peñalver et al. (2020) and Curcumin-PLGA prepared by Zakaria et al. (2022). It was observed that the uptake of CUR-LF NPs was stronger than that of free CUR. Two types of explanations could be proposed for this result. The first explanation assumes that LF coating contributes to the uptake of CUR-LF NPs by additionally initiating the LF receptor-mediated endocytic pathway (Zhao et al., 2018a), which was demonstrated by performing experiments with or without pretreatment with LF. With the pretreatment of LF, LF receptors on the surface of PC12 cells were bound and blocked, and CUR-LF NPs were competitively inhibited *via* LF receptor-mediated endocytic channels, resulting in reduced uptake in PC12 cells. In contrast, pretreatment with LF did not make a significant difference in the uptake of free CUR by PC12 cells. Therefore, the uptake of free CUR does not depend on LF receptor-mediated cellular endocytosis. The second explanation assumes that the positively charged nature of CUR-LF NPs facilitates binding to negatively charged cell membranes, and thus enhances the internalization of PC12 cells *via* charge-based interactions (Agwa et al., 2020).

Quantitative uptake was measured by HPLC (Figure 4B). We easily determined that the uptake of CUR-LF NPs by PC12 cells was higher than that of free CUR at each time point. The uptake rate of CUR-LF NPs was faster than that of free CUR in the first 60 min, as shown by the slope of the uptake curve. Overall, PC12 cells uptake in both groups was time-dependent. As the co-incubation time was extended, the uptake rate decreased, and the saturation trend gradually appeared.

### 3.6 Cytotoxicity assays

**FIGURE 5:**
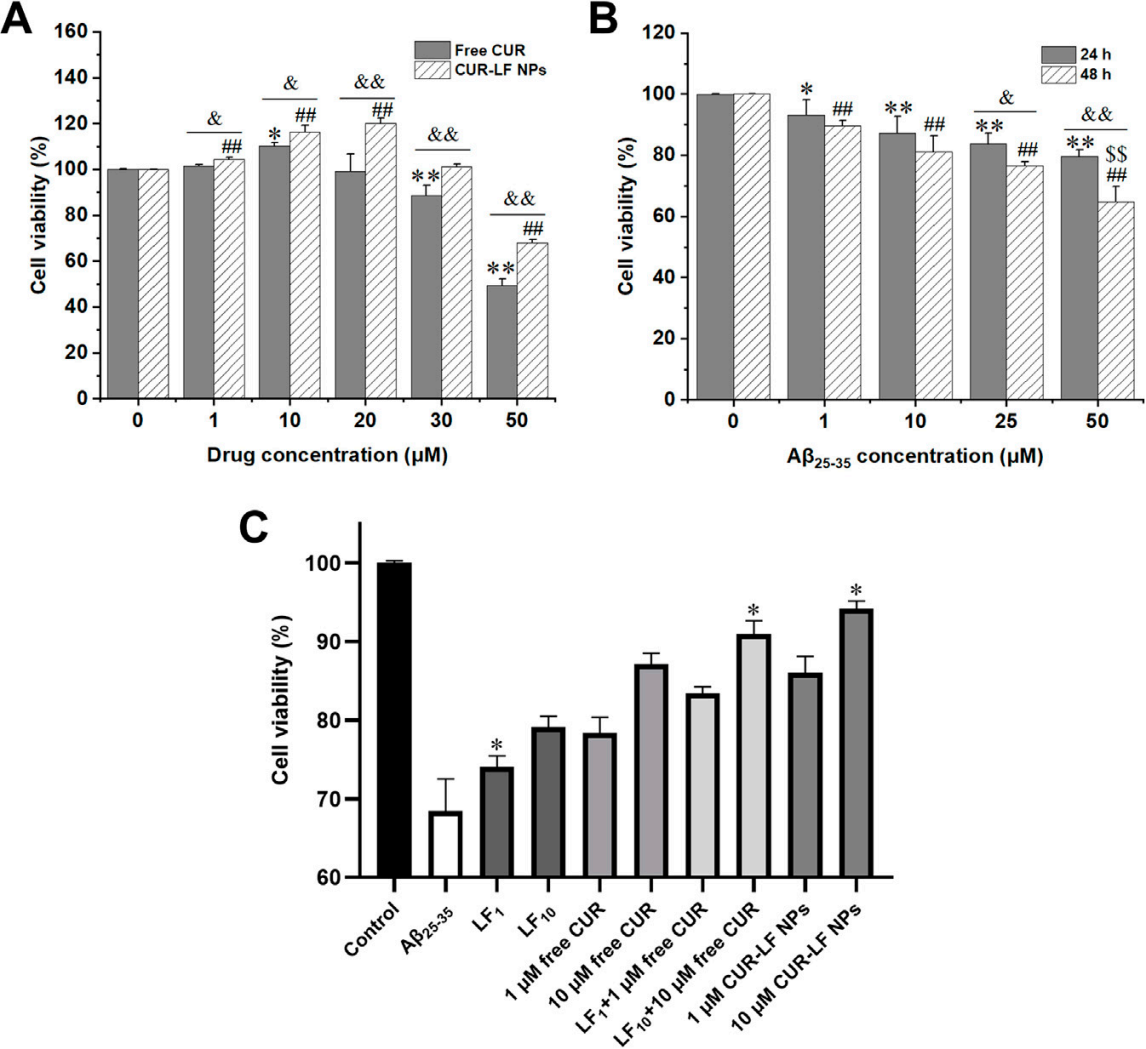
Cell viability assay **(A)** PC12 cells treated with free curcumin and CUR-LF NPs (1, 10, 20, 30, and 50 μM) for 24 h ^*^ *p* < 0.05, ^**^ *p* < 0.01 free curcumin group *versus* solvent control group; ^#^ *p* < 0.05, ^##^ *p* < 0.01 CUR-LF NPs group *versus* solvent control group; ^&^ *p* < 0.05, ^&&^ *p* < 0.01 free curcumin group *versus* CUR-LF NPs group **(B)** Investigation of induction concentration (1, 10, 25, and 50 μM) and time (24 and 48 h) of Aβ_25-35_-induced PC12 cell damage. ^*^ *p* < 0.05, ^**^ *p* < 0.01 Aβ_25-35_ group *versus* control group at 24 h induction time; ^#^ *p* < 0.05, ^##^ *p* < 0.01 Aβ_25-35_ group *versus* control group at 48 h induction time; ^&^ *p* < 0.05, ^&&^ *p* < 0.01 24 h *versus* 48 h induction time; ^$^ *p* < 0.01, ^$$^ *p* < 0.01 indicating the induction concentration and time was significantly different from other groups **(C)** Protective effect of lactoferrin nanoparticles, free curcumin, lactoferrin nanoparticles + free curcumin, and CUR-LF NPs on Aβ_25-35_-induced damage in PC12 cells. ^*^ *p* < 0.05, ^**^ *p* < 0.01 indicating the treatment group was significantly different from other treatment groups. Data were presented as mean ± SD (n = 3).

The potential cytotoxicity of CUR-LF NPs on PC12 cells was primarily evaluated using a CCK-8 assay and was utilized to ascertain the appropriate doses of CUR-LF NPs for further evaluation of their protective effect on Aβ_25-35_-induced PC12 cell damage. As shown in Figure 5A, free CUR did not exhibit cytotoxicity below 20 μM (cell viability >100%). Surprisingly, the safe dose of CUR was increased to 30 μM after encapsulation in LF nanoparticles. This could be attributable to the slow-release effect of CUR-LF NPs, which will not induce toxic reactions caused by excessive instantaneous drug concentrations. In addition, there were significant differences between free CUR and CUR-LF NPs at each concentration (1, 10, 20, 30, and 50 μM) (*p* < 0.05). It was clear that CUR-LF NPs displayed a better effect in promoting the proliferation of PC12 cells at concentrations of 1, 10, and 20 μM, and there was no significant difference in cell viability between 10 and 20 μM concentrations of CUR-LF NPs (*p* > 0.05). Therefore, in line with the principle of using as few drug doses as possible, 1 and 10 μM were selected as the low and high treatment doses of free CUR and CUR-LF NPs for the subsequent studies.

### 3.7 Protective effects of CUR-LF NPs on Aβ_25-35_-induced PC12 cells

The Aβ_25-35_-induced nerve damage model based on PC12 cells was constructed as previously described (Zhao et al., 2018b). From Figure 5B, it was found that the induction of 50 μM Aβ_25-35_ for 48 h could cause significant damage to PC12 cells to establish an appropriate *in vitro* AD model, and the cell viability under this condition was 68.4% ± 4.1%. Then, the protective effects of 1 and 10 μM free CUR and CUR-LF NPs against Aβ_25-35_-induced damage in PC12 cells were investigated. In addition, the protective effects of blank LF NPs (LF_1_, LF_10_) at different concentrations, whose LF content was equivalent to 1 and 10 μM CUR-LF NPs, were also evaluated in damaged PC12 cells.

As shown in Figure 5C, each formulation treatment group significantly improved the cell viability of Aβ_25-35_-induced damage in PC12 cells (*p* < 0.01). The protective capacity in order of magnitude was CUR-LF NPs > LF NPs + free CUR > free CUR > LF NPs and was dose-dependent (10 > 1 μM). Although LF NPs showed certain protective effects, the synergistic protective effect of simply mixing with free CUR was still lower than the equivalent dose of CUR-LF NPs in damaged PC12 cells. As mentioned in the cellular uptake studies, free CUR does not depend on the receptor-mediated endocytic pathway to access cells. Therefore, a simple mixture of free CUR and LF NPs does not promote the cellular uptake of free CUR, but merely superimposes their pharmacological effects. However, CUR-LF NPs not only had the synergistic pharmacological effects of CUR and LF, but also the LF coating increased the endocytosis of CUR, thus playing a stronger protective role. As a result, among all treatment groups, the 10 μM CUR-LF NPs group showed superior protective effects on Aβ_25-35_-induced damage in PC12 cells with cell viability of 91.0% ± 1.6%, and there was no significant difference between 1 μM CUR-LF NPs (86.1% ± 2.0%) and 10 μM free CUR (87.2% ± 1.3%) (*p* > 0.05). Therefore, 1 and 10 μM CUR-LF NPs were selected for further investigation.

### 3.8 Effect on antioxidant

**FIGURE 6:**
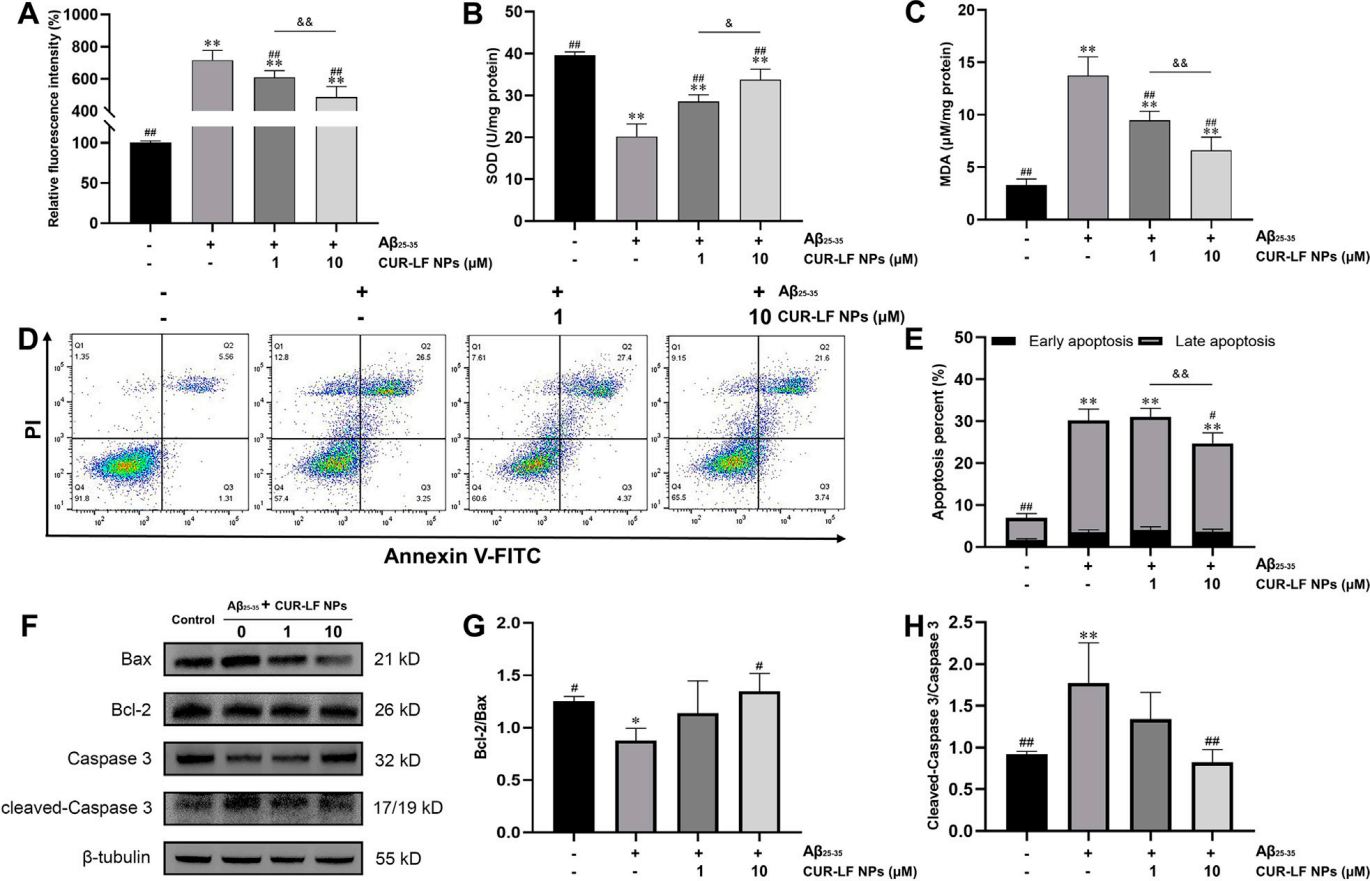
Protective effect of CUR-LF NPs on Aβ_25-35_-induced oxidative stress and apoptosis in PC12 cells **(A)** DCFH-DA fluorescence probe was used to detect intracellular ROS generation in PC12 cells **(B)** The intracellular SOD activity was measured using an SOD assay kit **(C)** The intracellular MDA level was evaluated with an MDA assay kit **(D)** Apoptosis of the treated PC12 cells was analyzed by flow cytometry using Annexin V-FITC/PI kit **(E)** Early and late apoptosis percent of PC12 cells **(F)** Representative western blotting images of Bax, Bcl-2, Caspase 3, cleaved-Caspase 3, and β-tubulin; Quantitation of Bcl-2/Bax ratio **(G)** and cleaved-Caspase 3/Caspase 3 ratio **(H)**. ^*^ *p* < 0.05, ^**^ *p* < 0.01 *versus* control group; ^#^ *p* < 0.05, ^##^ *p* < 0.01 *versus* Aβ_25–35_ group. ^&^ *p* < 0.05, ^&&^ *p* < 0.01 low dose treatment group (1 μM) *versus* high dose treatment group (10 μM). Data were presented as mean ± SD (n = 3).

The brain is highly sensitive to oxidative damage, and increased ROS in the brain could lead to excessive oxidation of proteins, lipids, and DNA, and reduce the activity of antioxidants such as glutathione, catalase, and SOD, thereby damaging neurons and even causing neuronal death (Ege, 2021). From Figures 6A–C, it was observed that the model group induced by Aβ_25-35_ significantly increased the generation of ROS from 100.4% ± 1.9% to 717.2% ± 60.2% and MDA from 3.3 ± 0.6 to 13.8 ± 1.8 μM/mg protein, and the content of antioxidant enzymes SOD significantly decreased from 39.5 ± 0.9 to 20.2 ± 3.0 U/mg protein compared to the control group. Adding low and high doses (1, 10 μM) of CUR-LF NPs to damaged PC12 cells greatly reduced the levels of ROS and MDA and improved the SOD activity, which was statistically significant compared to the model group (*p* < 0.05). There were significant differences between the 1 and 10 μM CUR-LF NPs treatment groups in all three indicators (*p* < 0.05), and the 10 μM CUR-LF NPs group showed better protective effects on Aβ_25-35_-induced oxidative stress in PC12 cells.

### 3.9 Effect on cell apoptosis inhibition

Aβ_25-35_ could induce apoptosis and affect the morphology of PC12 cells with rounding and wrinkling of the cells (Supplementary Figure S3). Flow cytometry analysis showed that Aβ_25-35_ induced apoptosis of PC12 cells, with the proportion of living cells decreasing from 91.6% ± 1.2% to 55.9% ± 3.2%, and the proportion of apoptotic cells increasing from 6.9% ± 1.4% to 30.2% ± 2.1% compared to the control group (Figures 6D, E). CUR-LF NPs (1, 10 μM) treatment significantly improved the proportion of living cells (60.7% ± 2.2%, 65.9% ± 3.0%) compared to the model group (*p* < 0.05), where 10 μM CUR-LF NPs significantly inhibited Aβ_25-35_-induced apoptosis, with the proportion of apoptotic cells decreasing to 24.7% ± 2.0% (*p* < 0.05).

Western blotting analysis was performed to elucidate the efficacy mechanism of CUR-LF NPs to protect against Aβ_25-35_-induced apoptosis in PC12 cells. The apoptosis genes Bax and Bcl-2 play an essential role in regulating the apoptotic process. Caspase 3, as their downstream apoptotic executor protein, could be activated to cleaved-Caspase 3 at the initiation of the apoptotic process (Chen et al., 2020). As shown in Figures 6F–H, Aβ_25-35_ significantly reduced the Bcl-2/Bax ratio of PC12 cells, activated Caspase 3 to cleaved-Caspase 3, and induced apoptosis compared to the control group (*p* < 0.05). In this study, low and high doses (1, 10 μM) of CUR-LF NPs upregulated the Bcl-2/Bax ratio and downregulated the cleaved-Caspase 3/Caspase 3 ratio, where the 10 μM CUR-LF NPs group could better reverse Aβ_25-35_-induced apoptosis in PC12 cells.

### 3.10 *In vivo* pharmacokinetic studies

**FIGURE 7:**
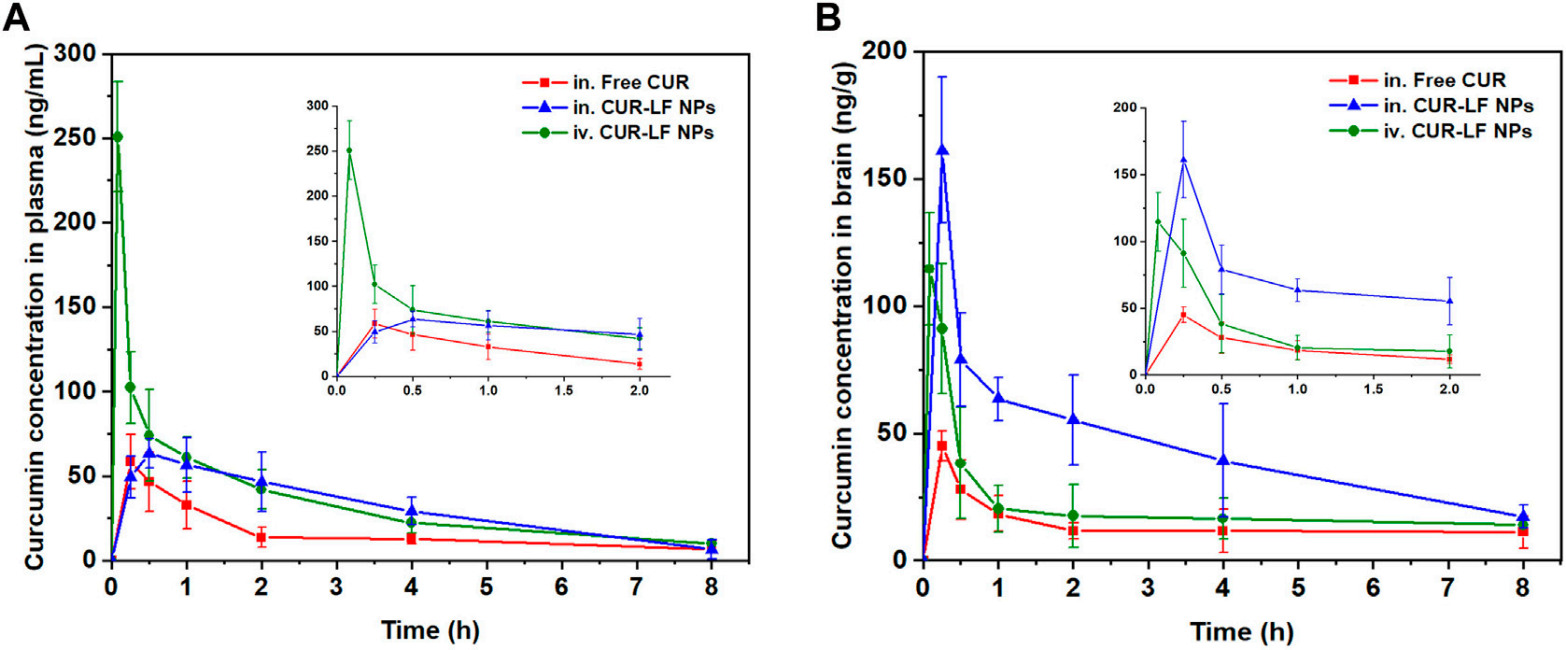
Concentration-time profiles of curcumin up to 8 h post-dosing in **(A)** plasma, **(B)** brain following intranasal administration of curcumin suspension and CUR-LF NPs, and intravenous injection administration of CUR-LF NPs at a dose of 10 mg/kg. Data were presented as mean ± SD (n = 3).

As shown in Figures 7A, B, we evaluated the pharmacokinetic profiles of CUR suspension, CUR-LF NPs *via* IN administration, and CUR-LF NPs *via* IV administration in plasma and brain at a dose of 10 mg/kg. The CUR concentrations were analyzed by UPLC-MS/MS, which was methodologically validated (Supplementary Figures S4, S5). The pharmacokinetic parameters, including AUC_0–8h_, t_1/2_, T_max_, and C_max_ were shown in Table 2. We clearly observed that the encapsulation of CUR in LF nanoparticles could prolong the elimination half-life in the systemic blood circulation and brain. The AUC_plasma_ of CUR-LF NPs was 2 times that of CUR suspension. In addition, the AUC_brain_ of CUR-LF NPs appeared to be 3 times that of CUR suspension. Overall, CUR-LF NPs could not only help CUR overcome defects, such as hydrophobic property and low permeability but also prolong the biological half-life and improve the bioavailability.

**TABLE 2:** The plasma and brain pharmacokinetic parameters after intranasal administration of curcumin suspension and CUR-LF NPs, and intravenous administration of CUR-LF NPs at a dose of 10 mg/kg. Data were presented as mean ± SD (n = 3).

Groups	Administration	Distribution	AUC _0–8h_ (ng/mL^a^h)	t_1/2_ (h)	T_max_ (h)	C_max_ (ng/mL)
CUR suspension	IN	Plasma	128.65 ± 29.30^b^	1.50 ± 0.23^b^	0.25 ± 0.00	58.40 ± 16.22
		Brain	108.90 ± 18.21^c^	2.00 ± 0.22^d^	0.25 ± 0.00	44.99 ± 5.95^c^
CUR-LF NPs	IN	Plasma	249.30 ± 30.37	2.35 ± 0.27	0.50 ± 0.00	63.31 ± 8.65
		Brain	352.91 ± 42.38	2.45 ± 0.13	0.25 ± 0.00	161.18 ± 28.71
CUR-LF NPs	IV	Plasma	292.72 ± 43.15	1.84 ± 0.37	0.08 ± 0.00	250.74 ± 32.63^b^
		Brain	167.00 ± 28.29^c^	1.49 ± 0.10^c^	0.08 ± 0.00	114.67 ± 21.98

^a^ *p* ‹ 0.05.

^b^ *p* ‹ 0.01 *versus* CUR-LF NPs, *via* intranasal administration in plasma.

^d^ *p* ‹ 0.05.

^c^ *p* ‹ 0.01 *versus* CUR-LF NPs, *via* intranasal administration in brain.

Meanwhile, CUR-LF NPs *via* IV administration were also evaluated for comparison with IN administration. Although AUC_plasma_ was considerably higher than AUC_brain_ of CUR-LF NPs *via* IV administration, it was reassuring to observe that a significant proportion of CUR-LF NPs could still cross the BBB into the brain because LF receptors were highly expressed in the BBB (Ye et al., 2013). The obtained results suggested that the brain accumulation of CUR *via* IN administration of CUR-LF NPs could be achieved mainly through two pathways: one was that CUR-LF NPs were absorbed into the peripheral circulation through the extensively vascularized respiratory area and then crossed the BBB *via* the LF receptor-mediated endocytic pathway, and the other was that CUR-LF NPs entered the brain *via* the direct nose-to-brain route (Tiozzo Fasiolo et al., 2021). For further studies, we calculated the BTE and DTP of CUR-LF NPs *via* IN administration by Equations 6, 7, which were 248.1% and 59.7%, respectively. The obtained results mean that the brain targeting of IN administration was higher than that of IV administration, and 59.7% of the CUR-LF NPs could be directly transported to the brain *via* the olfactory nerve pathway, olfactory mucosal epithelium, and trigeminal nerve.

## 4 Conclusion

Herein, we developed self-assembled CUR-LF NPs, which could be easily obtained by binding the hydrophobic region of LF to CUR without introducing cross-linking agents or toxic organic reagents. As a multifunctional glycoprotein, LF could be used as both a nanocarrier and targeting ligand in the nose-to-brain delivery of CUR-LF NPs. Moreover, it showed neuroprotective effects that could play a synergistic role with CUR. CUR-LF NPs could be favorably internalized by PC12 cells, thus playing an excellent role in protecting Aβ_25-35_-induced oxidative stress and apoptosis in PC12 cells. The *in vivo* pharmacokinetic results demonstrate that CUR-LF NPs could not only exhibit excellent brain accumulation *via* IN administration but also prolong the elimination half-life and improve the bioavailability of CUR. In conclusion, nose-to-brain delivery of CUR-LF NPs is a promising strategy for neuroprotection.

## Data Availability

The original contributions presented in the study are included in the article/Supplementary Material, further inquiries can be directed to the corresponding authors.
