# Supplementary material for: Nose-to-brain delivery of self-assembled curcumin-lactoferrin nanoparticles: Characterization, neuroprotective effect and in vivo pharmacokinetic study
Source: Front Bioeng Biotechnol. 2023 Mar 27;11:1168408. doi: 10.3389/fbioe.2023.1168408 (PMC10084992; doi:10.3389/fbioe.2023.1168408)
Supplement: Supplementary file 1 [file Table1.pdf]

Nose-to-brain delivery of self-assembled curcumin-lactoferrin nanoparticles: Characterization, neuroprotective effect and *in vivo* pharmacokinetic study

Linghui Li[1, †], Liwei Tan[2, †], Qian Zhang[1], Yushan Cheng[2], Yayuan Liu[2], Rui Li [1*], Shuguang Hou [1*]

*** Correspondence:**
Dr. Shuguang Hou, Tel/Fax: +86-28-61543118; E-mail: fansz1930@yahoo.com
Dr. Rui Li, Tel/Fax: +86-28-82606937; E-mail: lirui@cdutcm.edu.cn

1. Supplementary Figures and Tables

1.1 Supplementary Figures

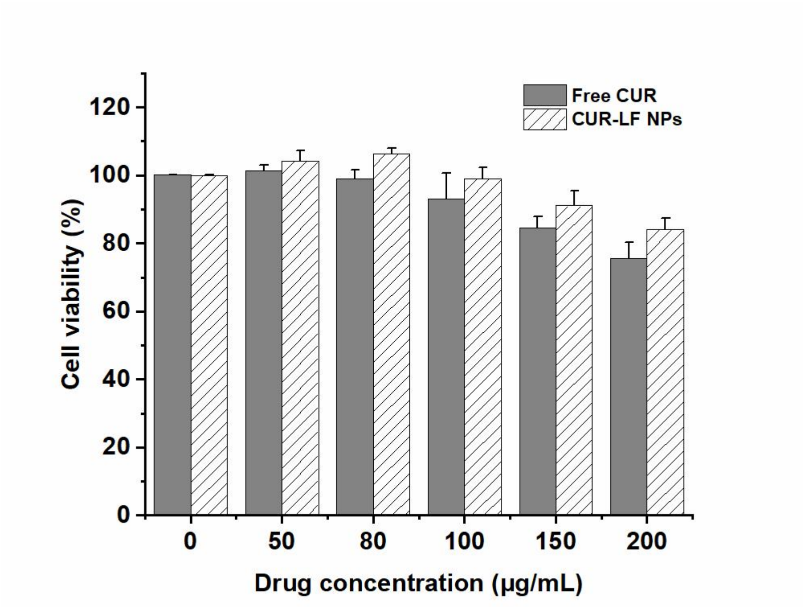

Supplementary Figure 1. Cytotoxicity assay of free curcumin and curcumin-lactoferrin nanoparticles (CUR-LF NPs) (50, 80, 100, 150, and 200 μg/mL) on MDCK cells after 4 h incubation. Data were presented as mean ± SD (n = 3).

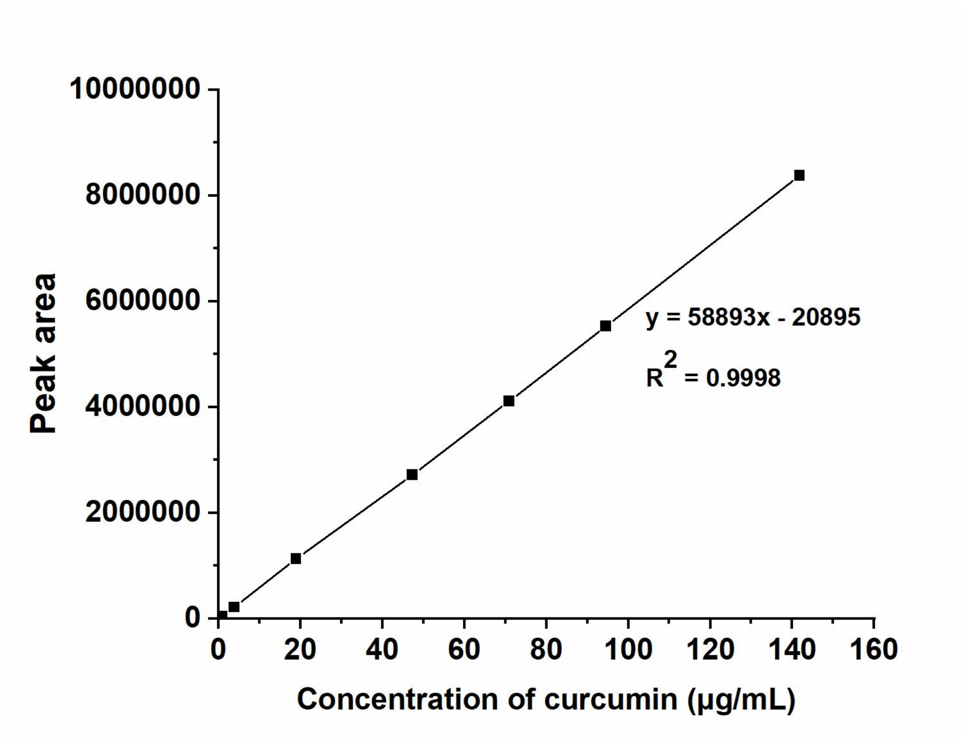

Supplementary Figure 2. The HPLC linear relationship of curcumin in the concentration range from 0.76 to 141.84 µg/mL.

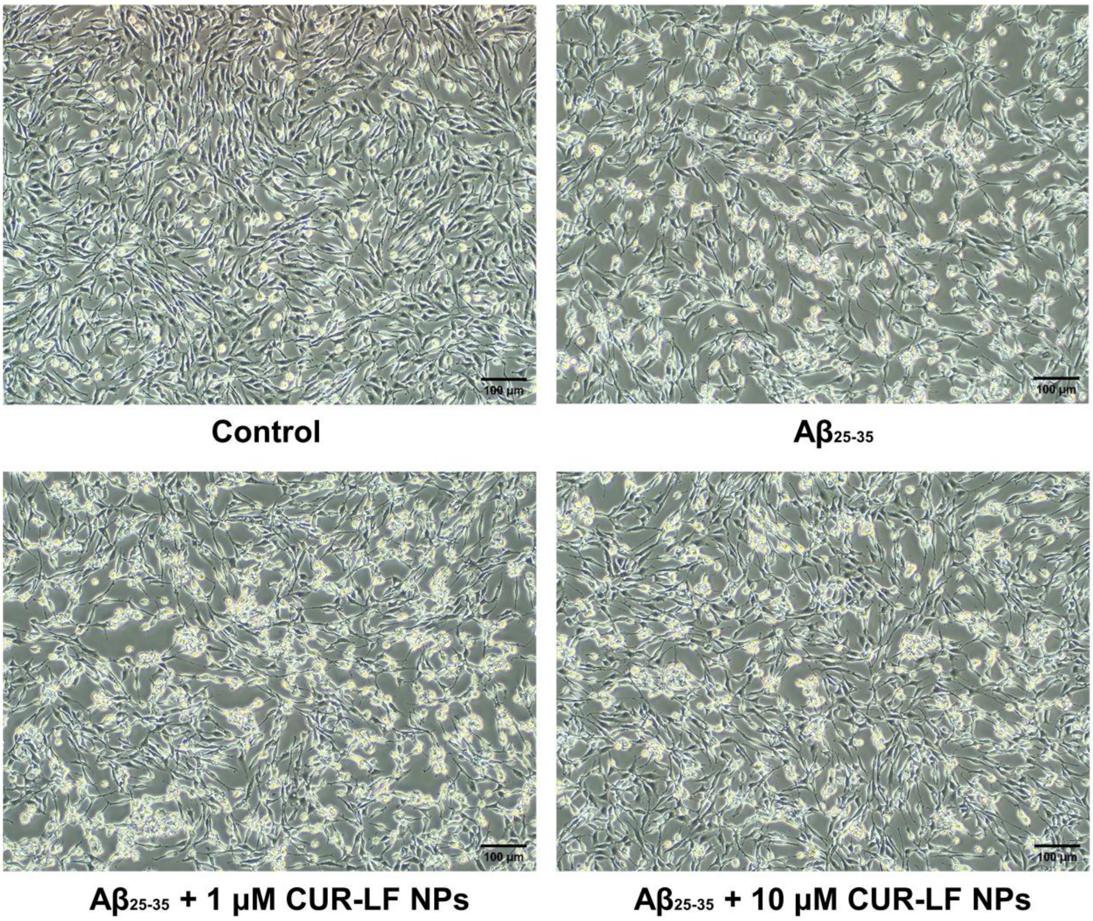

Supplementary Figure 3. Morphology of PC12 cells in different treatment groups.

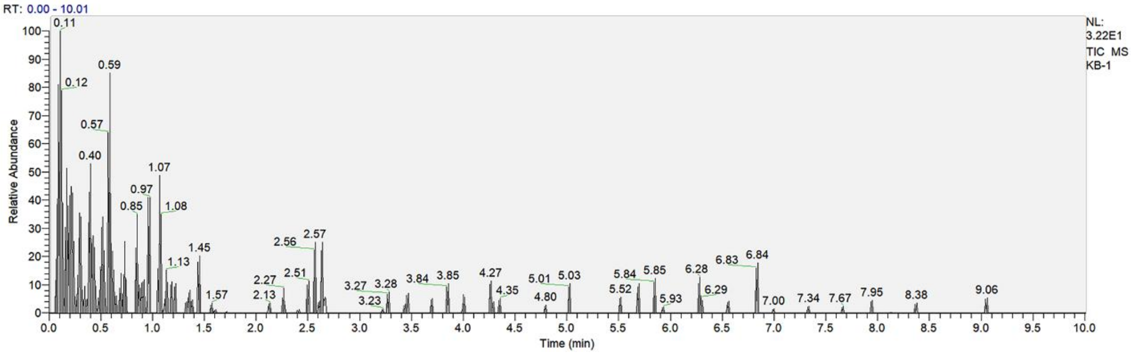

Blank plasma

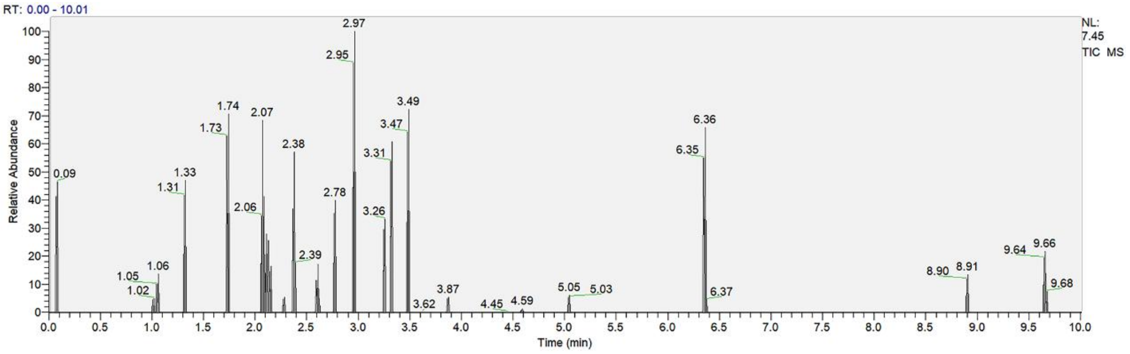

Blank brain tissue

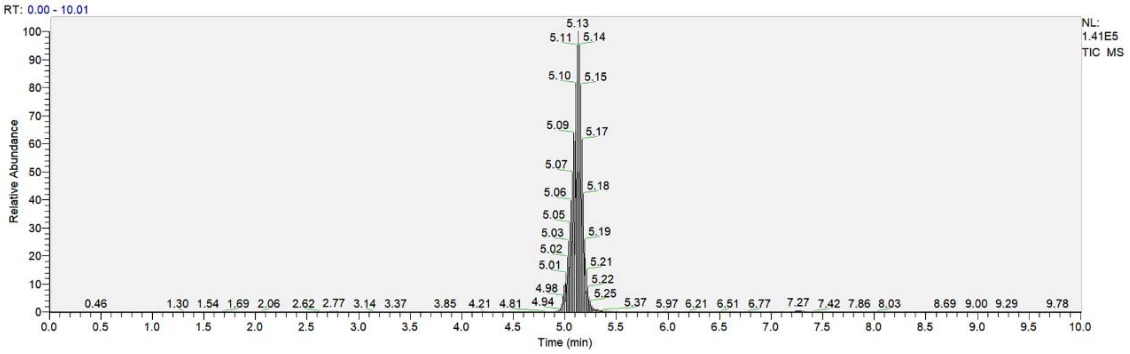

Curcumin standard

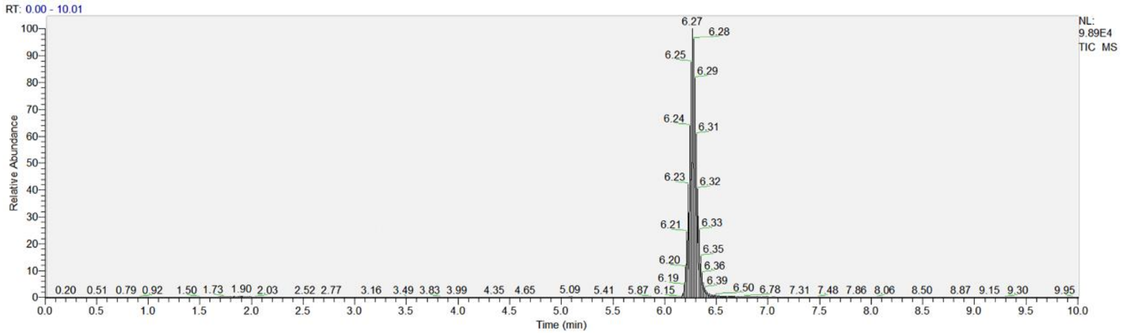

Magnolol standard

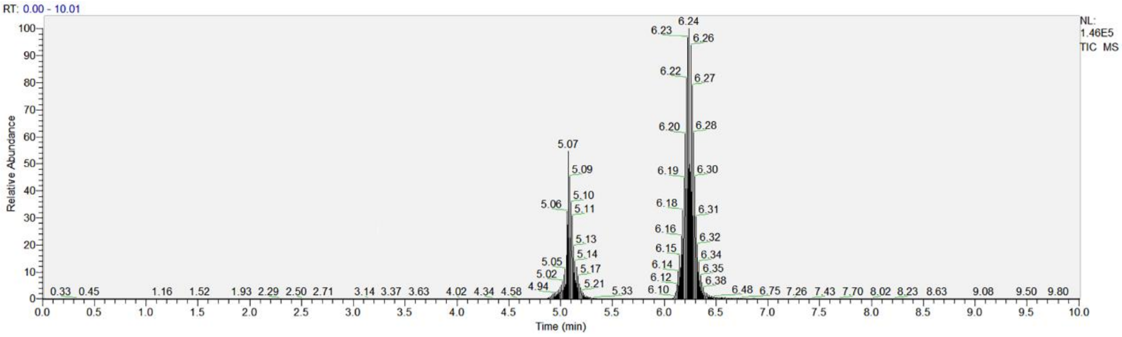

Curcumin and magnolol mixed standards

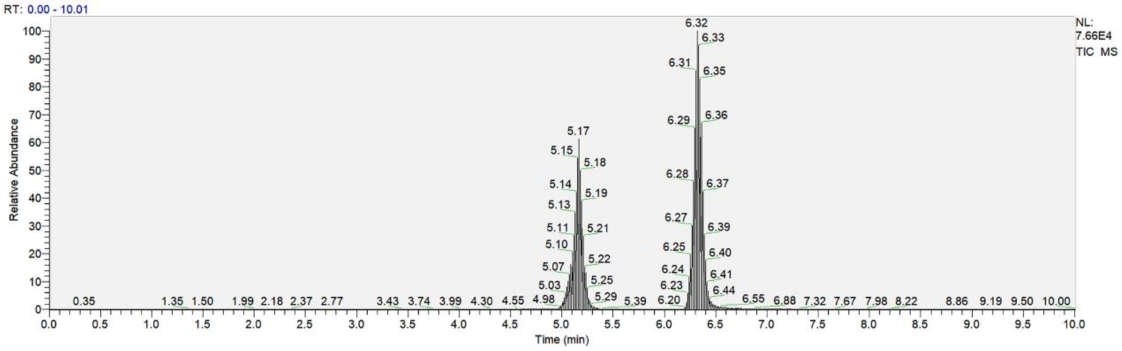

Curcumin and magnolol mixed standards in plasma

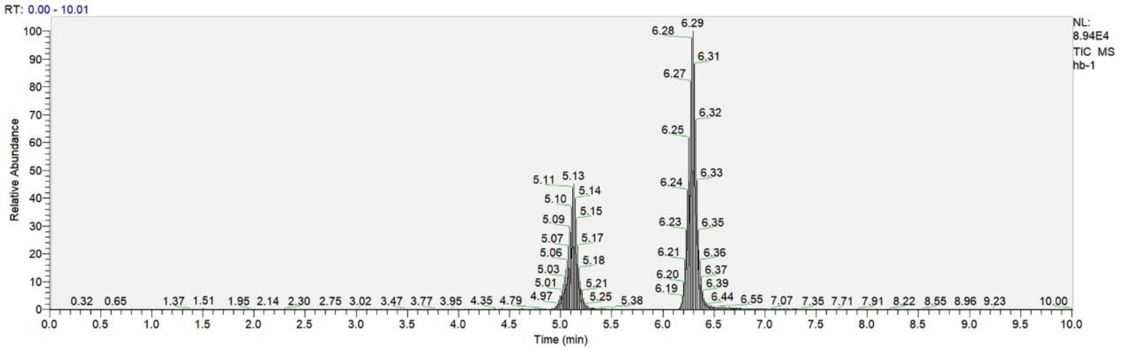

Curcumin and magnolol mixed standards in brain tissue

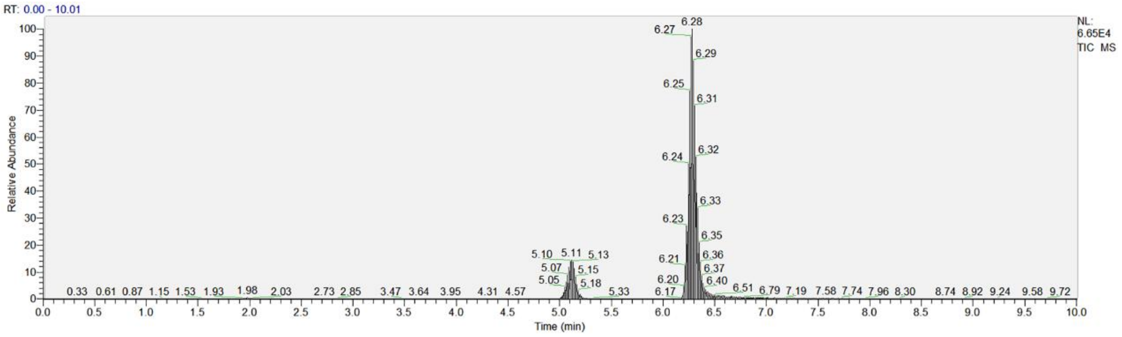

Curcumin and magnolol in plasma sample

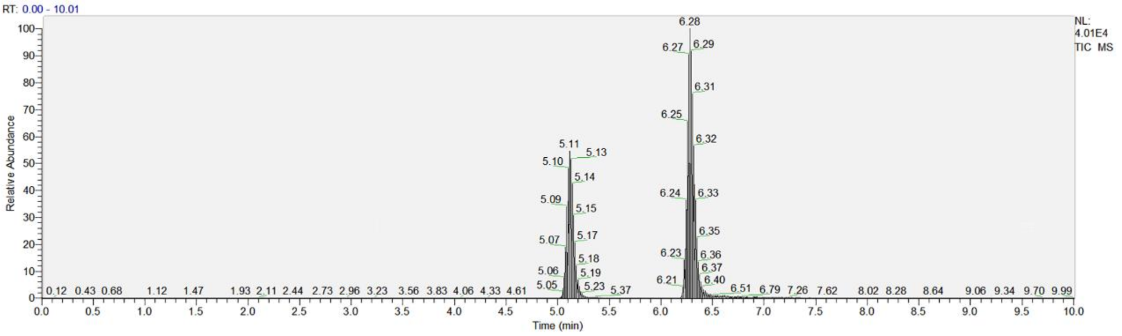

Curcumin and magnolol in brain tissue sample

Supplementary Figure 4. UPLC-MS/MS specificity examination of curcumin and magnolol in plasma and brain tissue**.**

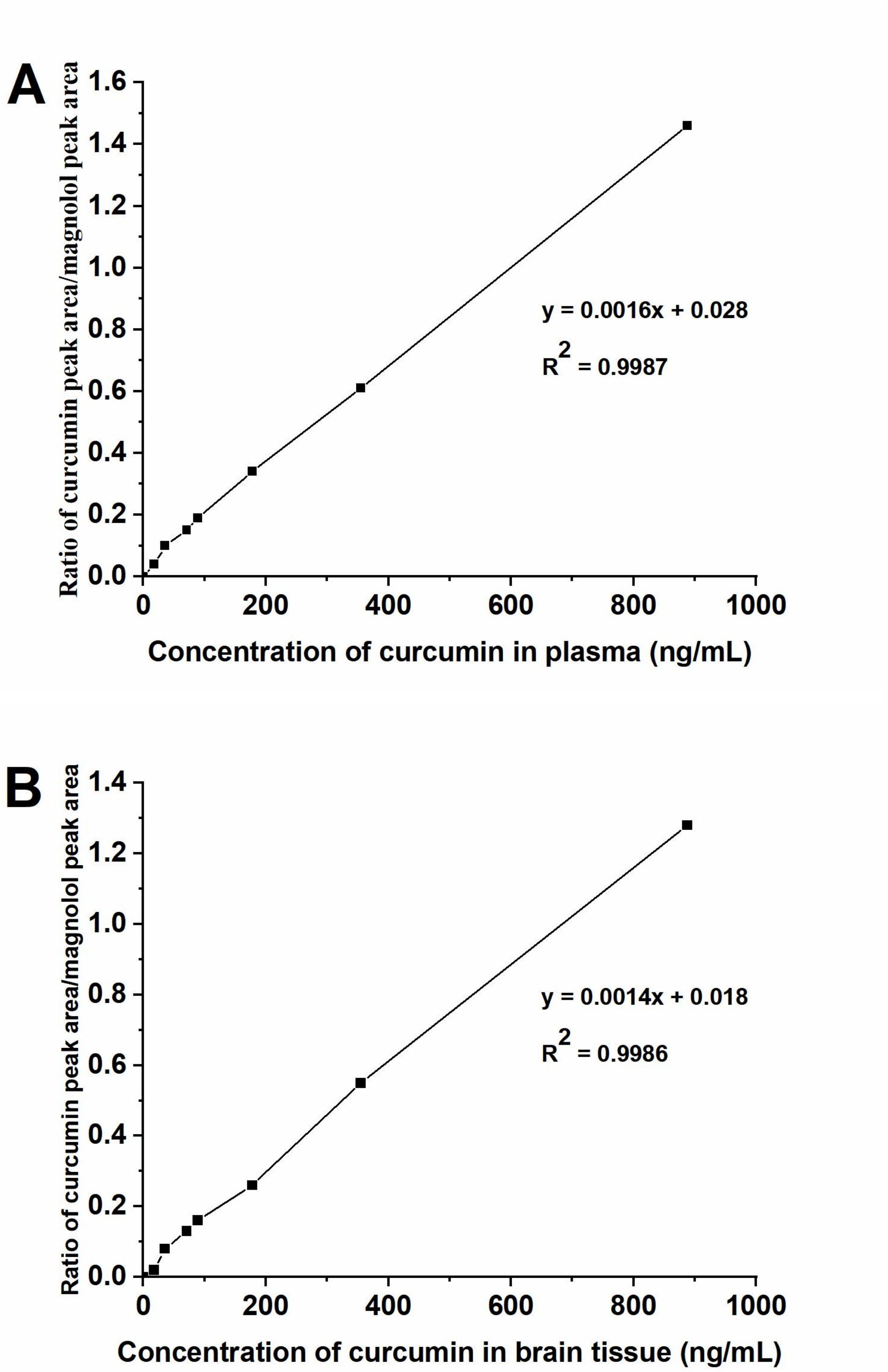

Supplementary Figure 5. The UPLC-MS/MS linear relationships of curcumin in plasma (A) and brain tissue (B) with the concentration range from 17.75 to 887.52 ng/mL.

1.2 Supplementary Tables

Supplementary Table 1. Fitting results of *in vitro* release of curcumin solution, curcumin suspension, and CUR-LF NPs.

	Model	Fitting equation	R^2
CUR solution	Zero-order equation	$Q=2.47t+43.13$	0.3386
	First-order equation	$Q=79.53(1-e^{-1.06t})$	0.9888
	Higuchi equation	$Q=16.03t^{1/2}+26.54$	0.6665
CUR suspension	Zero-order equation	$Q=2.34t+6.27$	0.8418
	First-order equation	$Q=57.12(1-e^{-0.11t})$	0.9972
	Higuchi equation	$Q=12.32t^{1/2}-3.95$	0.9651
CUR-LF NPs	Zero-order equation	$Q=2.38t+22.78$	0.5531
	First-order equation	$Q=58.77(1-e^{-0.50t})$	0.9783
	Higuchi equation	$Q=14.13t^{1/2}+9.33$	0.8436
